# Sustainable support solutions for community-based rehabilitation workers in refugee camps: piloting telehealth acceptability and implementation

**DOI:** 10.1186/s12992-020-00614-y

**Published:** 2020-09-15

**Authors:** Bria Mitchell-Gillespie, Hiba Hashim, Megan Griffin, Rawan AlHeresh

**Affiliations:** Occupational Therapy Department, MGH Institute of Health Professions, Charlestown Navy Yard, 36 1st Avenue, Boston, MA 02129 USA

**Keywords:** Community-based rehabilitation worker, CBR, LMIC, Refugee health, Telehealth, Telehealth implementation

## Abstract

**Background:**

The lack of training and education of Community-Based Rehabilitation (CBR) workers poses one of the most significant barriers to receiving effective occupational, physical and speech therapy for individuals with disabilities in Low-to-Middle Income Countries (LMIC), especially in countries with significant refugee populations. The aim of this study was to successfully implement a telehealth support system for CBR workers, evaluate the feasibility and acceptability of this intervention’s implementation among CBR workers in the CBR setting, and further identify strategies to address the deficit of skilled rehabilitation workers in LMIC through technological intervention.

**Methods:**

This pilot study included CBR workers and CBR managers to inform feasibility, acceptability, and sustainable implementation. The RE-AIM and Dynamic Sustainability Framework were incorporated to guide procedural design, survey development, data collection, data evaluation, and increase success of telehealth implementation. CBR workers participate in trainings, telehealth sessions, surveys and focus groups to inform feasibility and acceptability. CBR Managers participated in focus groups to inform feasibility and sustainable implementation. NVIVO 12 Software was utilized to develop themes from CBR worker and CBR manager responses.

**Results:**

Findings from this study demonstrate the need for additional training support for CBR workers in CBR settings throughout the entire treatment process. The telehealth system demonstrated successful short-term implementation across several domains of feasibility. Telehealth utilization was also proven acceptable, appropriate and necessary. Cultural beliefs, CBR worker training, and CBR Center infrastructure pose the most significant barriers to implementation of telehealth technologies in CBR Centers. CBR workers and managers confirmed the demand for future telehealth-based support systems, strengthening effort towards sustainability and scale-up.

**Conclusions:**

Telehealth can be utilized to support CBR workers that serve vulnerable and marginalized populations, and in turn improve the global health status among refugee populations by reducing inequitable access to quality health care. The results support the need for further research to rigorously evaluate effectiveness of telehealth interventions to support CBR workers.

## Background

Community-Based Rehabilitation (CBR) is a strategy designed by the World Health Organization (WHO) to overcome challenges to accessing rehabilitation services for individuals with disabilities in low-to-middle income countries (LMIC) [1]. CBR programs are implemented in more than 90 countries to disseminate rehabilitation services to individuals with physical and mental disabilities [2]. CBR workers are responsible for delivering these services and are typically trained to be generalists of rehabilitation in order to treat the wide range of disabilities they may encounter in their communities [3].

Studies delineate three levels of CBR workers: grass-root, mid-level and professional. Typically, grass-root workers are volunteers that may have several weeks of non-accredited training and mid-level workers have some form of accredited training to provide services without the supervision of a professional, supervise a grass-roots worker, and manage CBR programs [4]. Professionals have a formally accredited, certified degree and can be responsible for training and supporting mid-level workers through referral systems in which clients can attain more specialized services [4]. The literature suggests training mid-level workers in “professional” level capacities to address the deficit of trained rehabilitation professionals in LMIC yet acknowledges several ethical and procedural hindrances to implementing such comprehensive protocols [4]. Furthermore, studies recognize that initial training fails to address the on-going support CBR workers require [4]. The lack of specialty training and continuous service delivery support for CBR workers in LMIC is a barrier to comprehensive and effective rehabilitation intervention in CBR programs [5, 6].

The WHO currently estimates fewer than ten professional rehabilitation practitioners, including occupational, physical and speech therapists, per 1 million population in LMIC, although 80% of individuals living with a disability reside in LMIC [2]. In Jordan, the need for more specialized rehabilitation professionals in CBR programs is coupled with increasing prevalence of disability due to ongoing wars in neighboring countries, resulting in an influx of physically and mentally traumatized refugees [7]. The United Nations Relief and Works Agency (UNRWA) has managed ten CBR programs across ten refugee camps in Jordan since 1992, which provide access to health and rehabilitation services to refugees. Jordan is currently the world’s second largest host to forcibly displaced individuals [8]. The country’s demographics currently include 2.1 million Palestinian refugees, 660,000 Syrian refugees, 300,000 Iraqi refugees, as well as significant figures for Yemeni, Sudanese and Somalian refugees [7]. Jordan’s location, political and economic stability amidst surrounding violence has not only increased the total population in Jordan, but also applied pressure to the health care system overall, and thus the workers within the system responsible for carrying out comprehensive rehabilitation services to some of the world’s most vulnerable populations [7]. The increasing number of individuals with disabilities, and lack of specialized rehabilitation professionals, demonstrates the need for sustainable service delivery support of CBR workers in Jordan.

Telehealth technologies demonstrate significant potential to improve rehabilitation service delivery in LMIC, however, many studies in LMIC focus primarily on effectiveness of the telehealth intervention during the pilot stage [9, 10]. Sustainable implementation of telehealth systems is frequently obstructed by cost, time burden on clinicians, inadequate human resources, infrastructure, equipment legislation and funding [1, 5]. Factors impeding scalability of technological interventions in LMIC include complexity of the intervention, lack of technical consensus, health systems capacity, poor application of proven diffusion techniques, and lack of engagement of the adopting community [11]. Failure to robustly investigate feasibility and acceptability when designing and implementing telehealth systems has consistently limited the sustainable implementation and scalability of otherwise effective telehealth interventions in LMIC [2, 11].

Data regarding feasibility, acceptability, and appropriateness of utilizing telehealth interventions to support CBR workers in LMIC, especially in Jordan, is scarce. Further research is necessary to understand the reality of providing CBR workers with sustainable rehabilitation service delivery support through telehealth technologies. Therefore, the aim of this paper is to explore the feasibility and acceptability of implementing a telehealth system to support CBR workers in one refugee camp CBR center in Jordan. This article will also include key considerations for piloting telehealth interventions in CBR centers in Jordan with the intention to achieve sustainable implementation and scale.

## Methods

The following protocol was approved by the Partners Institutional Review Board (IRB) on September 6, 2019, submission number 2019P001266.

### Study design

The research team conducted a pilot study to assess the feasibility and acceptability of a telehealth support system implementation. Feasibility refers to the viability of the telehealth system in the context of a CBR center within a refugee camp, to support CBR workers. Acceptability encompasses the extent to which CBR workers consider the telehealth system to be appropriate, based on anticipated or experienced value of the intervention [12].

### Conceptual framework

**Fig. 1:**
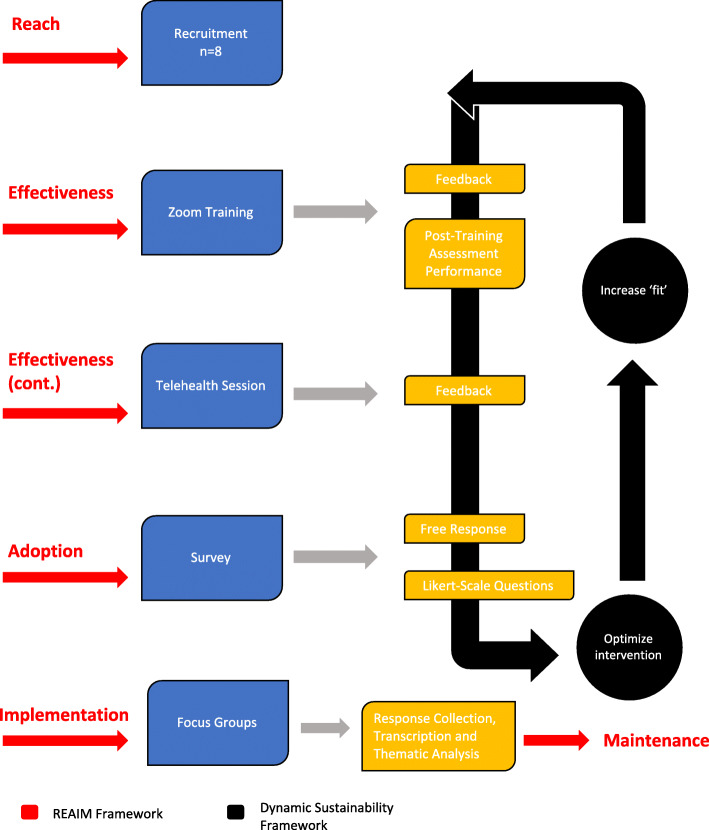
Use of RE-AIM and DSF

This study utilized the RE-AIM and Dynamic Sustainability Framework (DSF) (Fig. 1). The RE-AIM is recognized for assisting the transition of public health intervention pilots to real-world implementation through five sequential dimensions: Reach, Effectiveness, Adoption, Implementation and Maintenance [13]. Appropriate development of the first four dimensions is indicative of the intervention’s potential for the fifth and final stage of sustainable implementation: maintenance [14]. The RE-AIM framework was used to plan recruitment and training, use of the system, and develop survey and focus group questions that helped to holistically evaluate the feasibility and acceptability.

The DSF prioritizes sustainability through continuous integration and adaptation to progressively improve the ‘fit’ between the intervention and three multi-level contexts: intervention (telehealth system), practice setting (CBR center) and ecological system (policy, regulations, other practice settings) [15]. ‘Fit’ refers to the integration of innovations into health systems. The DSF was designed to understand how health intervention sustainability can be improved through implementation. The DSF informed the pilot procedure through continuous integration stakeholder feedback, in order to optimize the system and increase ‘fit’.

### Recruitment

The structure for CBR can vary significantly by program. In the Baqa’a Camp, CBR managers maintain relationships with government agencies, local authorities and centers, oversee facilitation of CBR strategy according to the CBR matrix, and manage CBR workers. CBR workers in this study were required to be affiliated with the Baqa’a Camp CBR Center in Amman, Jordan, fluent in Arabic or English, and presently treating individuals with physical or mental disabilities. CBR workers were recruited to use the telehealth system and inform the feasibility and acceptability. CBR managers were required to be affiliated with at least one refugee camp CBR Center, fluent in Arabic or English, and presently managing CBR workers or CBR sites. CBR managers were recruited to provide information on the feasibility of, and potential to sustainably implement, telehealth in CBR centers nationally.

**Fig. 2:**
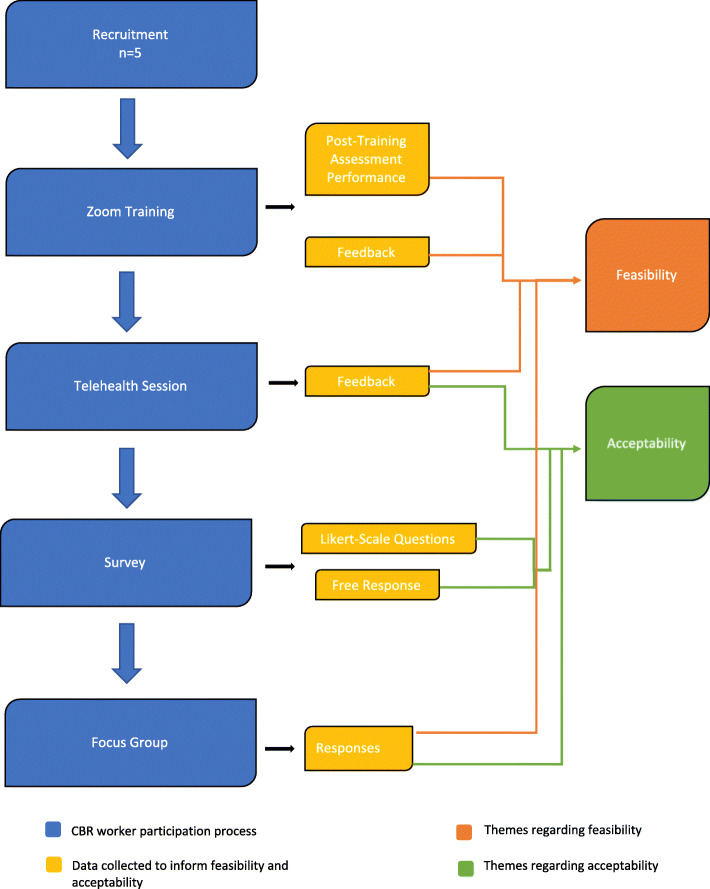
CBR Worker Participation

**Fig. 3:**
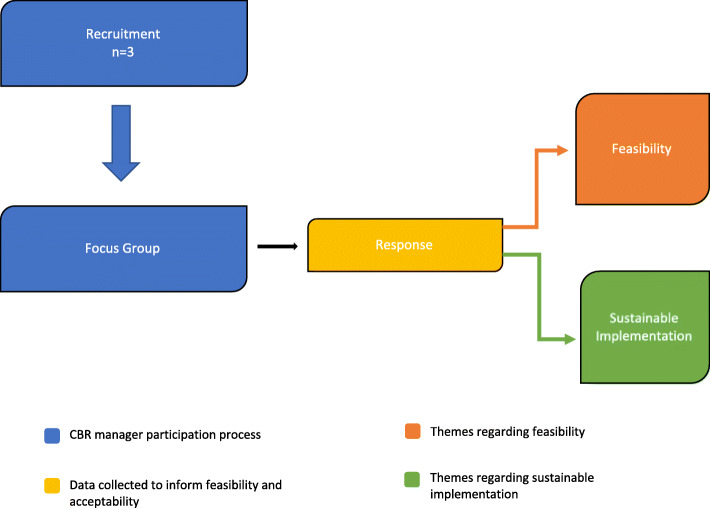
CBR Manager Participation

Participants were recruited via convenience sampling and informational flyers outlining study purposes and procedures, on-site at the Baqa’a Camp CBR Center. CBR workers were asked to participate in one system training session, at least one telehealth session, survey, and one focus group (Fig. 2). CBR managers were provided an informational handout outlining the study purpose and procedures and were required to attend one focus group (Fig. 3).

### Zoom training

Skype was tested primarily for implementation in the CBR Center. Skype Translator is a feature available via Skype that translates voice or text in real-time, which is desirable for providing assistance across a language barrier. Due to an inability to facilitate connection through skype within the CBR Center and other locations in Amman, Jordan, Skype was determined to be not feasible for supporting CBR workers and was excluded from further research procedure.

Zoom Video Communications software was used to evaluate the feasibility and acceptability of videoconferencing or chatroom-based telehealth sessions. Zoom was selected because of its user-friendly, easy to navigate interface and technical design, software customization, security encrypted video conferencing and data sharing. Although Zoom does not currently offer an Arabic interface, a picture-intensive training document was developed to guide the training protocol. The document featured a picture of each icon or menu item within the application alongside corresponding word description in Arabic explaining the function of the icon and its purpose relative to this study. The document was intentionally designed as a step-by-step guide to using Zoom for telehealth, and was made available for CBR workers to access after the training for use in telehealth sessions. CBR Workers used Zoom on an encrypted, HIPAA compliant iPad provided by the research team.

A 30-min onsite training occurred weekly for 4 weeks at the Baqa’a Camp CBR Center. The training protocol included a didactic component and post-training assessment delivered by two members of the research team. The didactic lesson covered the goal and rationale of the telehealth support system, device uses and procedures, shut down and startup of the system, customizations of the system, and troubleshooting common problems. The post-training assessment was used to assess participant competence as a new user and required participants to demonstrate opening the application, initiating video conferencing requests, initiating a chat room, connecting to audio and video. Scoring of the post-training assessment was determined based on successful or unsuccessful completion of five tasks: opening the application, selecting the correct icon to initiate a meeting, video calling a research team member, connecting to audio, and ending the call. A score of 80% (4/5 tasks completed correctly and unassisted) or better was necessary to participate in a telehealth session. The telehealth system was accessible to CBR workers for a one-hour block of time during a scheduled session, after completion of the training.

### Telehealth sessions

CBR workers in the camp were encouraged to participate in telehealth support sessions with the research team of two Doctor of Occupational Therapy students and one occupational therapy practitioner located in Boston, MA, as well as one physiotherapy practitioner located in Amman, Jordan. Three research team members were fluent in Arabic. CBR workers could arrange for assistance in sessions before client intervention (Type A), assistance during client intervention (Type B), or assistance after client intervention (Type C). At least two research team members discussed the client profile and health history prior to each telehealth session with the CBR worker. The research team recorded the session type, topic, real-time adaptations, modifications and feedback from CBR workers after each session. Feedback that recommended changes in training or sessions was considered by the research team and either adapted for the next session or remained unchanged due to technological or procedural constraints. The training protocol and telehealth sessions were used to assess CBR worker aptitude for utilizing telehealth and investigate the feasibility, acceptability, and barriers to receiving assistance through telehealth.

### Surveys

The purpose of the survey was to further identify the acceptability, demand and practicality of implementing a telehealth support system after having experience using the system. CBR workers completed surveys following telehealth sessions to inform acceptability based on user-perception of 1) the adequacy of support provided using the telehealth system, 2) preference to use the telehealth system in future sessions, 3) the appropriateness of the intervention in CBR work settings, 4) the ease of navigation for the telehealth system. The response options for the survey were based on a Likert-scale rating of one through five, representing strongly disagree, disagree, neutral, agree, strongly agree, respectively. The survey also included two prompts to suggestions for improvements and other comments that were used in the development of themes.

### Focus groups

The goal of the focus groups was to encourage participants to generate ideas about enhancing prospective telehealth system feasibility and acceptability and discuss improvements to be made in future applications for CBR settings in Jordan. There was one focus group for CBR workers and another for CBR managers. Topics related to technical, operational, economic, infrastructural and cultural feasibility and acceptability, and macro-level contextual factors such as policy, guidelines, and incentives were discussed. Potential barriers to sustainability and scalability of the intervention were also discussed in focus groups. The framework for the questions asked during the focus groups can be found in Additional file 1. The focus groups were audio recorded on a password protected and encrypted iPAD in a private room within the Baqa’a Camp CBR Center with the study participants, and three members of the research team.

## Data analysis

The training scores were assessed to determine readiness to participate in telehealth sessions. Feedback from each training was used to adapt and inform subsequent trainings and documented to contribute to the development of themes regarding feasibility. Telehealth session feedback was used to adapt and inform subsequent telehealth sessions and documented to contribute to the development of themes regarding feasibility and acceptability. Likert scale survey responses were analyzed as the average response score plus or minus the standard deviation to guide ‘fit’. Focus group responses were collected to inform themes of feasibility, acceptability and sustainability.

Data collected from the focus group, open-ended survey questions, training and session feedback was evaluated and organized using NVIVO 12 software. The audio recordings for the focus groups were transcribed and coded. Responses were developed into themes regarding feasibility and acceptability. The developed themes from the focus groups, survey questions and session feedback were dispersed through triangulation to achieve and confirm the accuracy of the data collected. The aim of this analysis was to identify user-focused perceptions and themes regarding feasibility and acceptability of a telehealth system supporting CBR workers in CBR Centers.

## Results

### Participants and demographics

Participants were recruited from December 2019–March 2020. Five CBR workers and three CBR managers participated in this study. All five CBR workers approached for recruitment elected to participate in this study. This total number accounted for 50% of CBR workers at the Baqa’a Camp. All three CBR managers approached for recruitment elected to participate in this study. The three CBR managers were affiliated with multiple CBR centers nationwide. The demographic information for the CBR workers and CBR managers that participated in this study is shown in Table 1 and Table 2.

**Table 1:** CBR Worker Demographics

CBR Workers (***n*** = 5)	CBRW1	CBRW2	CBRW3	CBRW4	CBRW5
**Educational Background**					
High School	X	X	X	X	X
Bachelor					
Master					
Other	Special Education Diploma				
**Work Setting**					
In home	X	X	X	X	X
CBR Center		X	X		
Other					
**Work Population**	Physical Disabilities	Communication and Special Education	Special Education/ Mild to Moderate Cognitive Disabilities	“All Disabilities”	“All kinds of disabilities”
**Training Level**					
Volunteer	X	X	X	X	X
Mid-Level					X
Professional					
Other					
**Age** #	30	47	36	24	23
**Gender**	F	F	F	F	F

**Table 2:** CBR Manager Demographics

CBR Managers (***n*** = 3)	CBRM1	CBRM2	CBRM3
**Current Professional Title**	Head at the Higher Committee of CBR Centers for the Disabled	Volunteer Supervisor of CBR workers	CBR Center Coordinator at the Higher Committee of CBR Centers for the disabled
**Educational Background**			
High School			
Bachelor	X	X	X
Master			
Other			
**Years of Experience** (#)	18	1	17
**Age** (#)	47	25	48
**Gender**	M	F	F

### Zoom training

Post-training assessment scores of CBR workers ranged between 80 and 100%. Participants had difficulty with remembering sequential steps for initiating a call, however, participants demonstrated 100% competence with the assistance of the provided training manual. All five trained CBR workers participated in the telehealth support sessions.

### Telehealth session feasibility

Providing assistance to CBR workers prior to session support occurred in 40% of telehealth sessions to help CBR workers plan and prepare treatment sessions (Type A). Assisting CBR workers during client session support occurred in 100% of telehealth sessions, indicating a substantial need for real-time support (Type B). Providing advice after client sessions for intervention modifications, discharge planning, parent education or home exercise program occurred in 60% of telehealth sessions (Type C). The telehealth support sessions for CBR workers included clients aged three to eight years old with musculoskeletal, communication, language, behavioral, social-emotional learning and cognitive disorders. Most CBR workers required assistance with effective interventions for motor skills, developmental delays and communication disorders, among other topics displayed in Table 3. Table 4 shows a summary of system and training challenges and their resolutions.

**Table 3:** Telehealth Session Topics

CBR Worker ID	CBRW1^**b,c**^	CBRW2^**a,b**^	CBRW3^**b,c**^	CBRW4^**b,c**^	CBRW5^**a,b**^
**Session Topic**	-Recommendation and demonstration of musculoskeletal stretches -Instruction for preparatory methods to therapeutic intervention -Education to parents and caregiver training -Developmental Delay	-Fine motor strengthening exercises -Fine motor activities to improve precision and coordination for age-appropriate Activities of Daily Living (ADLs) -Expressive and Receptive communication delay -Handwriting -Visual motor coordination -Behavioral attention	-Recommendations for flat foot -Weakness with lower extremities -Gross motor movement delay -Social-emotional learning -Improving dynamic standing balance to engage in ADLs and play	-Tactile and verbal cues to address drooling -Sensory Integration -Food aversion -Improve language comprehension -Learning difficulties -Parental education and training	-Considerations for working with clients with hearing impairments -Age-appropriate ADL performance Cognitive skills including memory and attention -Management of impulsive behaviors

^a^Assistance required BEFORE client intervention

^b^Assistance required DURING client intervention

^c^Assistance required AFTER client intervention

**Table 4:** System Challenges and Resolutions

System Challenges	Resolution
**Scheduling a meeting via email or text on Zoom**	This section was removed from the training protocol. Calls were spontaneous or scheduled outside of Zoom via Whatsapp.
**Wifi Connection**	Move to rooms nearest to the Wifi router. Run telehealth sessions exclusively in these rooms.
**Management of the iPad and client, simultaneously, during session**	Purchase of iPad kickstand or leaning the iPad against an appropriate set-up. Asking the parent to hold the iPad to focus on the child and therapist while they work together. Ensuring the availability of appropriate iPad set-up prior to future sessions.
**Language barrier**	Utilization of an on-site translator (CBR worker) and research team members fluent in Arabic and English
**Video time-outs**	Waiting a few moments for video to resume, ending and initiating the call again, moving nearer to the router.
**Zoom interface not in Arabic**	Picture intensive training manual guide was provided for the training session and for future use.
**Differences in user technological competence**	Additional training as necessary

### Surveys

The survey results indicated high rankings for the appropriateness of the intervention for CBR settings and workers, effectiveness of providing adequate support to CBR workers and desire for continued use of the system (Table 5). The scores for ease of navigation and minimal complication with the telehealth system were low during initial sessions due to early wifi complications, difficulty managing the iPad and need for more training, however increased in later sessions after modifications to the training and other specified challenges.

**Table 5:** Post-Session Survey Results

RE-AIM Domain	Question	CBRW1	CBRW2	CBRW3	CBRW4	CBRW5	Average	Standard Deviation
**Reach**	This telehealth system is appropriate for the setting(s) I work in as a CBR professional.	**5**	**5**	**4**	**5**	**5**	**4.4**	**0.89**
**Effectiveness**	This telehealth system adequately supported my needs in either treatment planning or intervention.	**5**	**5**	**5**	**5**	**5**	**4.8**	**0.45**
**Adoption**	I would like to use this telehealth system for assistance in future sessions.	**3**	**5**	**4**	**5**	**5**	**5**	**0.80**
**Implementation**	The telehealth system interface was easy to navigate, with minimal (less than 3) complications.	**2**	**3**	**3**	**5**	**5**	**2.8**	**1.34**

*1 = strongly disagree 2 = disagree 3 = neutral 4 = agree 5 = strongly agree

### Focus groups

#### CBR workers

##### Importance of training

CBR workers commented on the importance of having a training protocol to facilitate ease of implementation: *“As a group, we feel 80-95% comfortable using the system after just one training.”* Maintaining a training protocol in which only the features that would be consistently used were taught helped to simplify the system, making it easier to understand and adopt. Including a post-training assessment that provided hands-on, independent practice and gauged competency immediately after the training protocol seemed to reinforce skills learned during the training. CBR workers also agreed that proficiency navigating the application would be dependent on continued use of the system: *“We felt comfortable using the system, however, we believe we will gradually get better at using the system as we become more comfortable.”* One CBR worker mentioned that the addition of new features and modifications of existing features would warrant the need for further training sessions to restore feelings of competence in the system.

##### Appropriateness of the system

All CBR workers found the system to be most appropriate for assistance with assessing client conditions and providing insights for specific intervention ideas, protocols, service delivery, and justification for treatment due to their lack of training and experience: *“The system is helpful for assessment as [assessment] is not my specialty. I am not an occupational neither physiotherapist.”* CBR workers exchanged opinions regarding how the system addresses the lack of training and experience in their practice areas. One CBR worker highlighted a personal area in which she feels she requires more support: *“I need assistance reading and interpreting studies to apply scientific methods, and also documentation.”* The potential to access a support network of professionals was a frequently mentioned benefit of the system: *“I sometimes encounter problems and not sure what to do and it would be great to be connected to other professionals.”* Even the most formally educated CBR worker participating in this study expressed that receiving support on her client’s case was educational, beneficial, and improved her confidence. When asked about the appropriateness for CBR workers in other CBR Centers they have experience in, one worker replied: *“It’s the way you gave me ideas, no matter how good or experienced you are, you’ll encounter situations when you’re not sure what to do.”* Overall, CBR workers agreed that future telehealth support systems would need to be ongoing, as professional rehabilitation proficiency is not attainable for rehabilitation practitioners both in the Baqa’a Camp and other camps nationally.

Reflecting on the consequences to clients, CBR workers expressed feeling that clients would more quickly achieve therapy related goals due to the additional assistance of professionals through the system: *“Instead of a two-month treatment, it can be done in one month due to the right procedure taken right from the beginning.”* Following this sentiment, another CBR worker commented: *“Reducing effort is another benefit. Instead of doing trial-and-error for intervention, specialists on the system can suggest interventions that they know work.”* Other comments frequently mentioned the role of specialists in advising CBR workers through the system, specifically to *“give guidance, especially when something is done incorrectly.”*

##### Acceptability

Statements regarding potential widespread acceptability of telehealth among CBR workers occurred frequently: *“The session was wonderful. The system is easy to use. I wish I had this before. Other sites need to use this.”* Another worker recommended the research team *“Distribute this to all organizations and centers.”* When asked about the acceptability among other CBR workers, participants agreed that CBR workers in Jordan generally shared their struggles and would benefit from using this system, as well as enjoy the experience. Regarding use of telehealth to support CBR workers in other CBR centers and work sites in Jordan, CBR workers named common availability of internet access as a strength to the feasibility of telehealth in CBR settings: *“Every house has wi-fi and smart phones. The devices are cheaper now, so even low-income families can afford them.”*

##### The current referral system

The deficit in available specialists was discussed as a problem that affects CBR workers and the clients they serve: *“I have to place my clients on waiting lists for specialists and that can take weeks or months.”* Another sympathized with this frustration continuing, *“And sometimes the specialists don’t come.”* CBR workers shared experiences of placing their clients with more severe or acute needs on lengthy waiting lists. Due to scarcity of specialists and the long waiting times to see clients, all CBR workers expressed that having a support system that connects them with available specialists from a broader network is necessary and beneficial solution to the problems within the current referral system, and helping their clients receive quality care in a timely manner.

##### Barriers to continued use

CBR workers discussed some of the changes and new features they would like to have in prospective systems. One CBR worker highlighted that a weakness of the application was a lack of Arabic interface, which gained agreement by most of the other participants: *“It is better to have translation services that translates a sentence from Arabic to English immediately.”* A built-in translation service would provide easier communication for participants not speaking the same language.

When asked about features that would limit acceptability, one CBR worker mentioned concern regarding the potential presence of advertisements in future applications, as they would be distracting to treatment sessions and bothersome. CBR workers also discussed a topic expressed in survey feedback: managing the tablet during the sessions was difficult and distracting. Using an iPad during the session presented the challenge of maintaining clients and clinicians in an appropriate view while carrying out treatment sessions: *“I had a problem setting up the device. Fixing this will help in the session to avoid concentration issues.”* Two CBR workers mentioned this challenge when debriefing after the telehealth session. Not only did positioning the device present a challenge for CBR workers, it also made offering advice challenging for the specialists due to periodic inability to view specific client conditions being addressed and confirm the accuracy of treatments and stretches performed. Although no additional changes to the system and process were recommended at the time, participants agreed that more ideas would be generated regarding helpful adaptations with increased use. Evaluation of appropriateness and acceptability for telehealth in this setting will need to be ongoing.

Another potential barrier to implementation of telehealth to support CBR workers is parental perception and stigma: *“Parents might feel upset and insecure by filming their children; their privacy will be affected, and they fear it will be published.”* When asked to explain*,* CBR workers expressed that stigma around disability in Jordan might cause some parents to be concerned about the exploitation of their children, and suspicious about if and where session footage might be distributed. One CBR worker stated that although this stigma exists, *“This will be a great tool to support parents that want more specialized opinions.”* None of the parents that were approached refused their child’s role in this study due to telehealth utilization or otherwise. Nevertheless, CBR worker commentary indicates that stigma, parental knowledge and culture could initially be a barrier to utilization of telehealth in CBR settings.

#### CBR managers

##### The Baqa’a camp implementation

CBR Managers commented on the factors that made implementation feasible at the Baqa’a Camp in comparison to prospective feasibility at other camps they manage. One CBR manager reported that the Baqa’a Camp CBR center was the most *“technically and logistically advanced”*, which supported the technical and operational feasibility of this pilot. Managers frequently mentioned that this CBR center was able to adopt and implement this intervention due to superior organizational culture, center infrastructure and financial stability. The Head of the Higher Committee of CBR Centers for the Disabled stated that the Baqa’a Camp CBR Center was the main and leading center among others, highlighting that the CBR workers are more capable than other centers to embrace and utilize the telehealth system: *“In Al-Baqa’a, the workers are not just volunteers; they are specialized too, but in other centers they are mainly volunteers.”*

CBR managers agreed that implementing telehealth in the Baqa’a Camp CBR center was much easier than one could expect for implementing a similar program in other camps.

Furthering this point, this manager explained that the leading difference in workers is that CBR workers in the Baqa’a Camp were *“trained, mostly educated and well-chosen.”* to fulfill the CBR role. Another manager defined the Baqa’a Camp center as *“cooperative.”* When asked about necessary steps to initiate competence with this system in other camps through similar training protocols, this participant stated: *“It would be easy to implement in other CBR centers, but it is dependent on the education level of those in the center (CBR workers and parents) regarding electronic device use and the use of the app specifically.”* Another CBR manager explained that ability to apply rehabilitation advice provided via telehealth might also be a barrier to implementation in other camps, due to such varying knowledge and experience level among CBR workers across camps. CBR managers agreed that this discrepancy is more likely to be witnessed in CBR centers that exist in *“more remote areas”.*

##### Scaling to other camps

CBR managers confirmed CBR worker statements that internet access is common in most CBR centers in Jordan. One CBR manager of multiple CBR centers further explained that the quality of connection would vary by site, but overall internet access would not serve as a limiting factor to scaling telehealth across multiple sites. The issue of cost was also a commonly occurring topic regarding feasibility of implementing and maintaining this system in other camps. CBR managers agreed that some of the necessary tools for using telehealth in other CBR centers would be too expensive to purchase, as budgets vary significantly between camps. One manager explained that the benefit to using the system may not be clear to other CBR workers and managers without a similar pilot, and thus rationale for funding would be non-existent initially: *“We need tablets and wi-fi coverage, in the form of coupons for example, especially at the beginning to encourage them. They can depend on themselves after that.”* Regarding this matter, the same CBR manager reported that CBR centers would make an effort to independently fund and continue utilization of telehealth, after experiencing some of the benefits of the system that workers in the Baqa’a Camp have. CBR managers agree that if some initial funding of materials is provided, then individual CBR centers will be more willing to sustain the costs in their respective budgets.

CBR managers expressed that other CBR managers and personnel would be eager to adopt this system due to operational efficiency. Managers reported that the potential of telehealth to advance their practice, broaden their reach to the community and improve services of CBR workers would earn telehealth acceptability on behalf of CBR workers and managers: *“More and more people will be using it considering how beneficial it is for them.”* All managers viewed the system as appropriate for CBR settings, specifically for its role in providing necessary support to CBR workers, improving worker knowledge and effectiveness, and positively impacting clients. One manager specifically acknowledged the potential of telehealth to *“save effort and cover more patients.”* Another manager predicted the system would gain significant popularity over time.

##### Sustainable implementation

Managers collectively viewed telehealth as helpful, easy and vital to the advancement of the practice sites they manage: *“It is essential. We live in a world of technology, so necessary it will decrease time, effort, and cost.”* CBR managers reported no foreseeable negative impacts to using this system in Jordanian CBR centers. All reported anticipated significant growth in utilization by CBR workers for various reasons including the perceived and experienced benefits, the feasibility of telehealth to support CBR workers in centers, and the acceptability of the intervention.

Regarding political and governmental support, the CBR managers anticipate full support from organizations that fund and regulate CBR services: *“Once we use it and realize its benefits, I believe the Higher Board of the Disabled will encourage using it everywhere.”* Managers anticipate that as the benefits of telehealth become more well-known, and more pilots are completed, many members affiliated with CBR and in local communities will become vocal about using telehealth in this manner. One CBR manager stated that demonstrated benefit and effectiveness of a telehealth support system will be important to gaining support from funding and governmental agencies to sustain and scale this intervention: *“Once they understand the system and the need, it will be difficult for them not to support it.”*

### Triangulation

The triangulated themes are shown in (Table 6). One CBR worker disagreed with the theme on difficulty managing the iPad. Another emphasized that only a “small percentage” of individuals would be discouraged from participation in telehealth due to social stigma. The remaining themes were agreed upon by 100% of CBR workers and managers.

**Table 6:** Triangulated Themes

REACH	EFFECTIVENESS	ADOPTION	IMPLEMENTATION	MAINTENANCE
-Al Baqa’a Camp CBR Center has more skilled or better trained CBR Workers than other refugee camps, however the system is suitable for all camps due to its purpose to assist CBR workers in service delivery.	-The telehealth system would be beneficial to provide additional assistance with guiding treatment sessions, minimizing the need for referral to specialists, and serving as an additional resource to support CBR workers to plan treatment sessions or home programs.	-The telehealth system was easy to use.	-To maintain this system, government support is necessary.	-The iPad was difficult to manage while interacting with patients.
-Al Baqa’a Camp CBR Center has the appropriate skill, staff and infrastructure to support an online platform for telehealth.	-Specialists are not always available; this system would provide the support of a specialist more immediately.	-Having training for the telehealth system was beneficial and necessary. More training sessions will be necessary for continued use of the system.	-Obtaining government support for use of telehealth should be easy to obtain once the benefit of the system is demonstrated and resources are provided.	-Concern for stigma around disability may limit the use of telehealth in this setting.
-CBR workers may feel they are not specialized enough to manage a case and would like to connect with other healthcare professionals around the world to assist.	-The telehealth system will have a great impact on workers and patients.	-A system like this is essential, as we live in a world of technology. This system might decrease cost and time burden on clinicians.	-Some initial assistance with providing wifi and technology is necessary to continue the use of this telehealth platform in refugee camp CBR Centers.	-A system that has an Arabic language set-up or chatroom translator is necessary to improve the system.
-This telehealth system can be implemented in other CBR centers to support other CBR workers who would like additional support.	-The telehealth system may help CBR workers assist their patients in reaching their goals more quickly, reduce time and effort spent on ineffective intervention.	-CBR workers will feel more experienced and confident in using telehealth with increased use of the system.	-We feel we would be able to continue use of the system within this setting with minimal complication.	-The Wifi connection sometimes made use of the system difficult.

## Discussion

The goal of telehealth development and implementation in LMIC can be conflicting. A successful telehealth program meets the needs of the “users”, while also addressing an evidence-based need of a setting, community or country [16]. Additionally, pragmatic approaches to telehealth sustainable design, implementation and scale are of the upmost importance if telehealth is meant to be the optimal solution to resolve a given problem [16].

To our knowledge, this is the first study to explore the feasibility and acceptability of implementing telehealth to support CBR workers in Jordan. Thus, our findings have novel implications for future research initiatives and technological design regarding this population and setting. The telehealth support system was found to be feasible in this refugee camp CBR center, as findings from this study demonstrated the viability of a telehealth system and successful short-term implementation across several domains of feasibility. Telehealth was also found to be acceptable, as data collected from CBR workers and managers indicated that telehealth is desired for future use, provided adequate support, and is appropriate for CBR settings according to survey data ratings. The triangulated themes demonstrate CBR workers and managers agree that a telehealth system is necessary to improve care, provide support to strained CBR workers, and potentially reduce the amount of time and money spent working with clients. Participants anticipate telehealth systems to have a significant impact on their work roles and patient lives by reducing or eliminating the amount of time spent on ineffective interventions. According to the themes, some of the most significant barriers to more widespread implementation and adoption of this system include insufficient staff and infrastructure, difficulty navigating the complex application interface in a foreign language and supporting the initial cost of purchasing the materials. Additionally, stigma around disability in Jordan was named a limiting factor to continued use. This is due to the cultural beliefs of some regarding the origins and causes of disability, and fear of being exploited or shamed via an application-based system. Participants identified garnering government support for the telehealth system as a key solution to these barriers. CBR worker and manager comments in focus groups substantiated the decision to incorporate goals of sustainability and scalability throughout our pilot, further confirming the demand for a telehealth-based support system.

The RE-AIM and DSM frameworks were used to design a pilot study that addressed gaps in the literature related to implementation of innovative technologies and programs in LMIC. The incorporation of these frameworks proved essential, as both maintained the goal of sustainable implementation through focus on intervention planning (RE-AIM) and intervention optimization and ‘fit’ (DSF). The increasing ratings provided by CBR workers on surveys indicate that the DSF effectively helped to improve the ‘fit’ of telehealth in the CBR setting among CBR workers. The frameworks also promoted the collection of original insights for scaling among a broader target population and optimizing the application beyond the Zoom platform.

This pilot addressed previously mentioned barriers to sustainability and scalability.

through qualitative evaluation of technical, operational, infrastructural, cultural and economic feasibility [11]. Similar to other studies implementing technologies that are novel to the adopting community, a training to mitigate complexity of the intervention was planned and executed [11, 17]. Implementation science literature highlight the importance of technical consensus when planning for scalability [11]. The Zoom platform yielded a high degree of technical consensus as a simple platform that participants at the camp could easily learn to use, imagine optimizing to meet more of their needs and eventually scale-up. To further address technical consensus, infrastructural supports and barriers as well as potential burdens to implementing telehealth were evaluated.

When striving for sustainable implementation of telehealth in LMIC, focus on stakeholder perception, attitudes and needs is of as much priority as the technology itself [9, 11]. The adopting community was continuously engaged on two levels: 1) CBR workers as the system’s primary users and 2) CBR managers as the decision makers, institutional decision makers and implementation administrators in CBR centers. Identifying factors that influence engagement at an individual and institutional level was not only informative to cultural and operational feasibility, but also initiated collaborative discussions on impact and sustainability of this device from user and decision maker perspectives [9]. Low start-up costs and minimal resources required to implement this telehealth support system were an additional strength to this pilot’s sustained implementation. Finally, discussions regarding costs of implementation, potential equipment funding, garnering legislator support, health system capacity and human resources for sustaining and scaling telehealth interventions were facilitated in focus groups.

This study incidentally identified some specific diagnoses in which CBR workers feel they require more support, and whether assistance was most needed before, during, or after telehealth sessions, which carries its own implications for the training and support needs of CBR workers [4]. Similar to other studies completed in LMIC, this study also identified some cultural barriers and beliefs that might limit progression of technology use in LMIC, including stigma [17]. Additionally, the findings of this study mirror other mhealth studies in LMIC that highlight the importance of user perception as related to acceptability, providing training for users, ensuring low costs, and improving trust among users to support adoption and sustainable implementation [18, 19]. As found in similar studies, the complex relationship among use and degree of technology, contextual factors, human factors, and the broader operational ecosystem are substantial matters to cohesively incorporate in order to achieve sustainability of telehealth in CBR settings in Jordan [18, 20, 21].

Although Skype currently has the translation features desired by users, Zoom was ideal for this pilot, due to a superior connection for videoconferencing, which was an essential function for this pilot.

## Limitations

Our sample size was small and unbalanced between CBR workers and managers due to the COVID-19 pandemic and ethical obligation to cease recruitment; however, the pilot nature of this study provided invaluable finding to assist in building future more robust implementation investigations. All questionnaires and surveys were written in English and translated to Arabic. All written and spoken data collected from participants was then translated from Arabic back to English. As a result, there may be information that is incomplete, missing or lost in the translation process. Three members of the research team are fluent in both Arabic in English to minimize the impact of this limitation. CBR workers and managers were only recruited from one camp CBR center, therefore, findings are mostly applicable to this setting. The data collectively confirm the feasibility of telehealth utilization in the Baqa’a Camp CBR center, however, indicate that challenges to feasibility are likely to vary by CBR center due to variances in education levels of the target population. The research team provided the iPad with downloaded Zoom application. This presents a limitation to understanding the true costs and cost-effectiveness of telehealth implementation. Providing these materials allowed for the research team to quickly reach participants, as well as increase efficiency and consistency of use. This also supported efforts to represent low up-front costs for piloting this intervention, which allowed for actual use of the telehealth system to be included in the assessment of feasibility and acceptability. To compensate, the focus groups included questions regarding purchase of the appropriate technologies with candid discussion about costs and economic feasibility. Another limitation of this study was that surveys were not completed anonymously. This was done to allow the research team to identify the appropriate CBR worker to collaborate with in order to improve the ‘fit’, based on their responses.

## Conclusion

This pilot study sought to understand the reality of providing sustainable telehealth support to CBR workers in Jordan. The research team achieved this goal by identifying and recruiting a target population of CBR workers that was representative of CBR workers nationally in qualifications, experiences and roles. This study highlights the impact of, and ability to use, telehealth to support CBR workers. The representativeness of this CBR center according to settings nationwide, the willingness of current and prospective CBR workers to participate in a telehealth pilot, procedures for continuing the program and motivating or deterring factors for adopting telehealth in CBR settings was also assessed. Participants expressed fidelity to use of a telehealth support system in the future. This study also highlighted adaptations made throughout the pilot as well as implementation strategies for success. Telehealth has demonstrated feasibility and acceptability to address the deficit of professional rehabilitation practitioners in at least one CBR center in Jordan. With these findings, telehealth has the potential to address the gap in specialized rehabilitation care received by refugees through providing support for CBR workers in Jordan.

Further studies should investigate the effectiveness of telehealth interventions in supporting CBR workers, and continued evaluation of alternative solutions optimal to address the deficit in skilled rehabilitation workers in LMIC. Additionally, further studies should consider assessing feasibility and acceptability in other CBR centers.

## Supplementary information

**Additional file 1.** Framework for focus group questions

## Data Availability

Data supporting the results of this article can be obtained through email of the corresponding author, Bria Mitchell-Gillespie: bmitchellgillespie@gmail.com
