# Supplementary material for: Sustainable support solutions for community-based rehabilitation workers in refugee camps: piloting telehealth acceptability and implementation
Source: Global Health. 2020 Sep 15;16:82. doi: 10.1186/s12992-020-00614-y (PMC7491020; doi:10.1186/s12992-020-00614-y)
Supplement: Supplementary file 1 — Additional file 1. Framework for focus group questions [file 12992_2020_614_MOESM1_ESM.docx]

**Additional file 1**

**FRAMEWORK FOR FOCUS GROUP QUESTIONS**

CBR WORKERS

- What factors would encourage future participation?
- What features impact engagement?
- Is the target population suitable for using this intervention?
- How representative is this target population or CBR Center?
- Was the support system useful in treatment planning and/or intervention?
- What are the challenges of using this system?
- What are the benefits of using this system?
- Did the intervention meet the expressed needs?
- How could the system better meet the expressed need?

CBR MANAGERS

*Inquire about various types of feasibility* (economic, operational, cultural, managerial, infrastructural)

- Did the intervention fit the mission and purpose of the CBR center?
- Why would you choose to continue use of this system?
- Why would you choose to not continue to use this system?
- What adaptations or modifications will be necessary to help implement the initiative to fit the different settings (CBR Centers nationally)?
- What characteristics of this specific CBR center made implementation suitable or unsuitable?
- Any personal motivating or deterring indications of implementing the system for use daily? Nationally? With specific populations or diagnoses? With workers in other sites?
- What would be necessary (support/resource) for this CBR Center to continue a program like this as independently as possible?
